# Early and current physical activity: relationship with intima-media thickness and metabolic variables in adulthood

**DOI:** 10.1590/bjpt-rbf.2014.0040

**Published:** 2014

**Authors:** Manoel C. S. Lima, Maurício F. Barbosa, Tiego A. Diniz, Jamile S. Codogno, Ismael F. Freitas, Rômulo A. Fernandes

**Affiliations:** 1 Programa de Pós-Graduação em Ciências da Motricidade, Instituto de Biociências, Universidade Estadual Paulista (UNESP), Rio Claro, SP, Brazil; 2 Grupo de Investigação Científica Relacionada a Atividade Física (GICRAF), Laboratório de Investigação em Exercício (LIVE), Departamento de Educação Física, Universidade Estadual Paulista (UNESP), Presidente Prudente, SP, Brazil; 3 Programa de Pós-Graduação em Radiologia, Departamento de Radiologia e Diagnóstico por Imagem, Universidade Federal de São Paulo (UNIFESP), São Paulo, SP, Brazil

**Keywords:** maintenance of physical activity, physical activity, atherosclerosis, insulin resistance, movement

## Abstract

**Background:**

It is unclear whether early physical activity has a greater influence on intima-media thickness and metabolic variables than current physical activity.

**Objective:**

To analyze the relationship between current and early physical activity, metabolic variables, and intima-media thickness measures in adults.

**Method:**

The sample was composed of 55 healthy subjects of both sexes (33 men and 22 women). Total body fat and trunk fat were estimated by dual-energy X-ray absorptiometry. Carotid and femoral intima-media thickness were measured using a Doppler ultrasound device. A 12-hour fasting blood sample collection was taken (fasting glucose and lipid profile). Early physical activity was assessed through face-to-face interview, and the current physical activity was assessed by pedometer (Digi-Walker Yamax, SW200), which was used for a period of seven days.

**Results:**

Current physical activity was negatively related to total cholesterol (rho=-0.31), while early physical activity was negatively related to triglycerides (rho=-0.42), total cholesterol (rho=-0.28), very low density lipoprotein (rho=-0.44), and carotid intima-media thickness (rho=-0.50). In the multivariate model, subjects engaged in sports activities during early life had lower values of very low density lipoprotein (b=-8.74 [b=-16.1; -1.47]) and carotid intima-media thickness (b=-0.17 [95%CI: -0.28; -0.05]).

**Conclusion:**

Early 95%CI physical activity has a significant influence on carotid intima-media thickness, regardless of the current physical activity.

## Introduction

In the last decades, the occurrences of obesity and cardiovascular diseases have increased among adults^1^. Similarly, the burden of these outcomes on healthcare costs has increased dramatically^2^. It is recognized that obesity and metabolic variables are related to the increase in the intima-media thickness of blood vessels^3^^-^ 5. Therefore, in clinical settings, vessel intima-media thickness has been used as a tool to screen for vascular injury^6 ^and cardiovascular diseases^7^. Moreover, greater carotid intima-media thickness (CIT) is related to stroke, myocardial infarction^8^, and atherosclerosis^9^^,^ 10. 

In contrast, greater current physical activity has been associated with lower mortality and increased life expectance^11^, as well as lower CIT^12^^,^ 13. In hypertensive subjects, physical activity attenuates CIT progression, which is explained in part by the reduction in cardiovascular risk factors^14^. 

It has been documented that early physical activity (its maintenance during childhood and adolescence) seems to prevent cardiovascular and metabolic outcomes in adulthood^15^^,^ 16. However, it is unclear whether early physical activity has a greater influence on intima-media thickness and metabolic variables than current physical activity. Thus, the purpose of this study was to analyze the relationship between current/early physical activity, metabolic variables, and intima-media thickness measures in adults. 

## Method

### Subjects

The present study includes the baseline measures of a cohort study "Maintenance of physical activity throughout life and vascular stiffness in adults: cross-sectional analysis and cohort of 12 months", which is being conducted in Presidente Prudente, SP, Brazil (from 2013 to 2014). In this cohort study, the following inclusion criteria were adopted: (i) age between 30 and 50 years; (ii) no previous history of stroke or myocardial infarction; (iii) no amputation or visual problems related to diabetes mellitus (there were no diabetic subjects); (iv) no physical limitation that would affect physical activity; (v) classification as either persistently active (at least one year of supervised sports practice outside school in both childhood [age 7 to 10] and adolescence [age 11 to 17]) or persistently sedentary (no supervised sports practice outside school in both childhood and adolescence); and (vi) informed consent. 

In the selected sample, there were two hypertensive subjects under medical treatment (beta blocker), while 18.2% (n= 10) reported the use of either statins or other medication. Among subjects with either hypertension or dyslipidemia there was no simultaneous use of two or more drugs (combination therapy). In additional analyses, there was no significant relationship between medicine use and the metabolic/cardiovascular variables and, therefore, these subjects were maintained in the sample. 

The researcher responsible for the cohort study contacted fitness clubs of the city and also contacted the Human Resources Sector of the University to identify and contact eligible candidates to participate in the cohort. In the presented study, the sample was composed of 55 subjects of both sexes who fulfilled all inclusion criteria and, thus, were invited to participate in this study. All study procedures were approved by the Ethical Research Group of Universidade Estadual Paulista (UNESP), Presidente Prudente, SP, Brazil (approval nº 173.571/2012), and all subjects signed an informed consent form. 

### Anthropometry and body composition

Body weight was measured using a digital scale (Filizola, PL-200, to the nearest 0.1 Kg) and height was measured with a wall-mounted stadiometer (to the nearest 0.1 cm) with a maximum length of 220 cm (Sanny, Standard ES2030). Body mass index (BMI [Kg/m^2^]) was calculated by the use of body weight and height values. Waist circumference was measured in centimeters (cm) using a metallic tape. 

All anthropometric measures were made in a reserved room following standard methods. 

Total body and trunk fat were estimated by a Dual-Energy X-ray Absorptiometry (DEXA) scanner (Lunar DPX-NT; General Electric Healthcare, Little Chalfont, Buckinghamshire, UK), version 4.7. All participants were in light clothing, with no shoes or metal belongings on the body, and in supine throughout the examination (approximately 15 minutes). Total body and trunk fat were expressed in percentage values by the DEXA software. All measurements were carried out at the University laboratory in a controlled temperature room. Each morning, before the beginning of the measurements, the DEXA was calibrated by the same researcher, according to the references provided by the manufacturer. 

### Intima-media thickness measurement

To measure the carotid and femoral intimamedia thickness (CIT and FIT, respectively), we used a Doppler ultrasound device (Toshiba Xario, SSA-660A). All tests were performed at a hospital in morning (between 8 and 11 am), and analyses were performed by a medical doctor specializing in diagnostic imaging. The procedures adopted for the examinations followed the recommendations of the Brazilian Society of Radiology^17^. Prior to the examination, all subjects were at rest, lying in a supine position in a quiet room and acclimatized. To measure CIT, the neck was positioned in hyperextension and slightly inclined at 45º. The measurements were taken from the posterior wall of the artery, farthest from the transducer, manually and with the caliper method. Three measurements of CIT and FIT were obtained in the stretch of 15 mm free of plaques, where the pattern is clearly observed. For analysis of the results, we considered the mean values in millimeters (mm) of each artery. 

### Blood samples

All blood sample collections and biochemical analyses were done in a private laboratory, which meets the standardization criteria of quality control adopted by the Brazilian Health Ministry. A 12-hour fasting blood sample collection was taken. Samples were collected in vacuum tubes containing gel with anticoagulant. Then the blood was centrifuged for 10 minutes at 3,000 rpm. To measure fasting glucose, total cholesterol (TC), triglycerides (TG), very low density lipoprotein cholesterol (VLDL-C), low density lipoprotein cholesterol (LDL-C), and high density lipoprotein cholesterol (HDL-C), an enzymatic colorimetric kit processed in an Autohumalyzer A5 unit was used^18^. 

To obtain glycated hemoglobin (HbA_1C_), blood was collected under vacuum in sealed tubes containing ethylenediaminetetraacetic acid (EDTA) as lyophilized anticoagulant. The determination of the glycated hemoglobin was performed in primary tube by high performance liquid chromatography (HPLC) equipment in D10 -Hemoglobin A1C Testing System (Bio-Rad(r), France). HPLC is a standardized methodology for HbA_1C _determination, certified by the National Glycohemoglobin Standardization Program (NGSP) traceability of analytical performance with the reference methods Diabetes Control and Complications Trial (DCCT) and United Kingdom Prospective Diabetes Study Group (UKPDS)^19^^,^ 20. 

### Early and current physical activity

Early physical activity was assessed using two questions^15^^,^ 16: (i) "Outside school, did you engage in any organized/supervised sport activities in clubs for at least 1 year between the ages of 7 and 10 years?" and (ii) "Outside school, did you engage in any organized/supervised sport activities in clubs for at least 1 year between the ages of 11 and 17 years?" Other physical activities, such as dance modalities (e.g. ballet), were also included. Subjects were classified as persistently active (sports practice in both childhood and adolescence [n=45]) or persistently sedentary (no sports practice in both childhood and adolescence [n=10]). 

Current physical activity was assessed by pedometer (Digi-Walker Yamax, SW200). The pedometer was fixed laterally at the hip and taken off only during periods of sleep, activities in the pool, and during shower. The pedometer was used for a period of seven days. At the end of each day, the subjects recorded the number of steps taken throughout the day. In the morning, to begin collecting the data, the "reset" button was pushed to zero out the equipment. The mean values of steps in the week were assigned as the level of current physical activity, and the sample was stratified as either physically active (≥10,000 steps/day [n=21]) or sedentary (<10,000 steps/day [n=34])^21^. 

### Potential confounders

Face-to-face interview was used to assess the regular use of anti-hypertensive drugs, lipid-lowering drugs, and anti-diabetic drugs (statistical analysis was carried out using the number of drugs reported). Additionally, resting systolic and diastolic blood pressures (SBP and DBP, respectively) were measured at rest in a seated position after at least 10 minutes of rest. Medicine use and blood pressure measures were used as potential confounders, and multivariate models were adjusted by both variables. 

### Statistical analysis

Several variables presented non-parametric distribution and, thus, median and interquartile range (IR) were used as descriptive statistics. Mann-Whitney's test compared fat, metabolic, and cardiovascular variables according to current and early physical activity. Spearman's rank-order correlation (rho) analyzed the relationship between CIT/FIT and independent variables (Spearman's rank order correlation was used even in dataset with normal distribution). Finally, the linear regression model was used (expressed as beta values [b] and 95% confidence interval [b]), in which 95%CI the statistically significant relationships in the multivariate model were adjusted simultaneously for potential confounders (sex, age, body fat, blood pressure, medicine use, and trunk fat). All analysis were performed by the statistical software BioEstat (release 5.0 [BioEstat, Tefé, Amazonas]), and the statistical significance was set at *p*-value <0.05. 

## Results

The sample was composed of 33 men and 22 women (Table 1). In the overall sample, the prevalence of insufficient current physical activity was 38.2% (n=21) and only 18.2% (n=10) of the sample reported no sports practice during childhood and adolescence. Regarding current physical activity, only body fat (*p*-value=0.025) and trunk fat (*p*-value=0.046) were statistically different. Total cholesterol had marginal significance (*p*-value=0.050). 

**Table 1:** General characteristics of the sample stratified by current physical activity (Brazil, n=55).

Variables	≥10,000 steps/day (n=21)	<10,000 steps/day (n=34)	*p*-value
	Median (IR)	Median (IR)	
M/F	14 / 7	19 / 15	
Age (years)	37.9 (6.3)	38.9 (12.4)	0.132
Body weight (Kg)	77.6 (12.5)	74.9 (19.9)	0.856
Height (cm)	173.5 (11.1)	174.2 (18.7)	0.829
BMI (Kg/m^2^)	24.5 (6.2)	24.6 (5.3)	0.822
WC (cm)	80.5 (11.7)	82.3 (15.6)	0.456
Body fat (%)	23.1 (9.5)	29.6 (10.3)	0.025
Trunk fat (%)	27.8 (8.3)	33.1 (12.1)	0.046
Glucose (mg/dL)	89.6 (9.9)	89.3 (8.5)	0.842
Triglycerides (mg/dL)	77.1 (43.6)	98.1 (72.2)	0.194
Total cholesterol (mg/dL)	184.7 (49.6)	203.5 (58.8)	0.050
HDL-C (mg/dL)	50.8 (9.3)	53.1 (16.1)	0.253
LDL-C (mg/dL)	118.4 (42.1)	130.1 (40.6)	0.163
LDL-C/HDL-C (mg/dL)	2.34 (1.2)	2.34 (1.2)	0.579
VLDL-C (mg/dL)	15.4 (8.7)	19.6 (14.4)	0.103
HbA_1c_ (mmol/mol)	5.3 (0.5)	5.3 (0.6)	0.747
CIT (mm)	0.60 (0.09)	0.66 (0.14)	0.078
FIT (mm)	0.48 (0.06)	0.51 (0.10)	0.138
Steps/day	11,906.1 (1718)	8,286.1 (2136)	0.001
SBP (mmHg)	110 (20)	111 (20)	0.810
DBP (mmHg)	80 (10)	81 (10)	0.529

M = male

F = female

BMI = body mass index

WC = waist circumference

**HDL-C:** = high density lipoprotein cholesterol

**LDL-C:** = low density lipoprotein cholesterol

**VLDL-C:** = very low density lipoprotein cholesterol

**HbA1C:** = glycated hemogloblin

CIT = carotid intima-media thickness

FIT = femoral intima-media thickness

SBP = systolic blood pressure

DBP = diastolic blood pressure.

Adults who engaged in sports practice during childhood and adolescence had lower body fat (*p*-value=0.001), trunk fat (*p*-value=0.001), triglycerides (*p*-value=0.002), total cholesterol (*p*-value=0.036), and VLDL-C (*p*-value=0.001) than non-engaged adults (Table 2). Moreover, CIT was significantly lower in persistently active adults (p-value=0.001). Current physical activity was also increased in adults engaged in sports practice during childhood and adolescence (*p*-value=0.001). 

**Table 2:** General characteristics of the sample stratified by maintenance of sport activity during childhood and adolescence (Brazil, n=55).

Variables	Persistently Active (n=45)	Persistently Sedentary (n=10)	*p-*value
	Median (IR)	Median (IR)	
M/F	29 / 16	4 / 6	
Age (years)	38.3 (6.9)	43.8 (13)	0.121
Body weight (Kg)	77.3 (17)	74.8 (17.5)	0.639
Height (cm)	174.1 (12.8)	166.3 (18.5)	0.015
BMI (Kg/m^2^)	24.3 (4.8)	26.1 (9.4)	0.190
WC (cm)	80.5 (13.2)	88.5 (21.5)	0.210
Body fat (%)	24.1 (10.5)	40.2 (17.6)	0.001
Trunk fat (%)	31.3 (8.2)	47.9 (14.6)	0.001
Glucose (mg/dL)	89.3 (9.1)	91.1 (11.5)	0.413
Triglycerides (mg/dL)	76.2 (46.9)	127.7 (36.8)	0.002
Total cholesterol (mg/dL)	190.1 (47.8)	225.4 (54.2)	0.036
HDL-C (mg/dL)	52.5 (12.3)	50.1 (19.9)	0.793
LDL-C (mg/dL)	123.2 (40.2)	132.3 (38.2)	0.385
LDL-C/HDL-C (mg/dL)	2.3 (1.2)	2.7 (1.3)	0.789
VLDL-C (mg/dL)	15.2 (9.3)	25.5 (7.4)	0.001
HbA_1c_ (mmol/mol)	5.3 (0.5)	5.3 (0.8)	0.637
CIT (mm)	0.60 (0.11)	0.78 (0.11)	0.001
FIT (mm)	0.49 (0.08)	0.53 (0.12)	0.287
Steps/day	9,476.1 (3148)	6,848.8 (4474)	0.001
SBP (mmHg)	110 (20)	105 (30)	0.920
DBP (mmHg)	80 (10)	75 (20)	0.885

M = male

F = female

BMI = body mass index

WC = waist circumference

**HDL-C:** = high density lipoprotein cholesterol

**LDL-C:** = low density lipoprotein cholesterol

**VLDL-C:** = very low density lipoprotein cholesterol

**HbA1C:** = glycated hemogloblin

CIT = carotid intima-media thickness

FIT = femoral intima-media thickness

SBP = systolic blood pressure

DBP = diastolic blood pressure.

Regarding medicine use, lowering lipid drugs was negatively related to FIT (rho=-0.30; p-value=0.024), but not CIT (rho=-0.11; p-value=0.412). Metabolic variables were not related to medicine use. Current physical activity was negatively related to TC (rho=-0.31). On the other hand, early physical activity was negatively related to TG (rho=-0.42), TC (rho=-0.28), VLDL-C (rho=-0.44), and CIT (rho=-0.50) (Table 3). 

**Table 3:** Relationship between current/early physical activity, intimal thickness, and metabolic variables in adults (Brazil, n=55).

Physical Activity	Glucose	TG	TC	HDL-C	LDL-C	LDL-C/HDL-C	VLDL-C	HbA_1c_	CIT	FIT
	Rho	rho	rho	Rho	Rho	rho	rho	rho	rho	rho
Current	0.01	–0.22	–0.31*	–0.20	–0.19	–0.02	–0.24	–0.07	–0.26	–0.02
Early	–0.11	–0.42^§^	–0.28*	–0.03	–0.11	–0.03	–0.44^§^	–0.06	–0.50^§^	–0.08

* = p-value <0.05

§ = p-value <0.01

Rho = Spearman's rank correlation

TG = triglycerides

TC = total cholesterol

**HDL-C:** = high density lipoprotein cholesterol

**LDL-C:** = low density lipoprotein cholesterol

**VLDL-C:** = very low density lipoprotein cholesterol

**HbA1c:** = glycated hemogloblin

CIT = carotid intima-media thickness

FIT = femoral intima-media thickness

In the multivariate model, regarding current physical activity, the number of steps was negatively related to total cholesterol (b=-0.004 [b=-0.007; 95%CI-0.001]) regardless of sex, age, and body composition (Table 4). The same patterns had been identified among adults engaged in sport activities during early life, when considering VLDL-C (b=-8.74 [b=-16.1; -1.47]) and CIT (b=-0.17 [95%CI: 95%CI-0.28; -0.05]). 

**Table 4:** Adjusted relationship between current/early physical activity, carotid intimal-media thickness and metabolic variables in adults (Brazil, n=55).

Relationship between variables		Model – 1	Model – 2
Independent	Dependent	β (β_95%CI_)	β (β_95%CI_)
Physical Activity			
Current	TC	–0.004 (–0.007; –0.001)	–0.004 (–0.007; –0.001)
Physical activity			
Early	TG	–42.6 (–89.2; 3.94)	–28.7 (–85.8; 28.3)
Early	TC	–26.3 (–50.4; –2.21)	–25.2 (–56.1; 5.56)
Early	VLDL-C	–10.1 (–16.4; –3.87)	–8.74 (–16.1; –1.47)
Early	CIT	–0.18 (–0.26; –0.10)	–0.17 (–0.28; –0.06)

TG = triglycerides

TC = total cholesterol

**VLDL-C:** = very low density lipoprotein cholesterol

CIT = carotid intima-media thickness

**Model-1:** = crude

**Model-2:** = adjusted by sex, age, general, trunk fat, medicine use, systolic and diastolic blood pressures

95%CI = 95% confidence interval

## Discussion

This was a cross-sectional study in which early and current physical activity were related to cardiovascular and metabolic outcomes. It identified that early physical activity seems to be a determinant factor in the occurrence of increased cardiovascular risk, regardless of current physical activity. 

In the present study, current physical activity was negatively related to total cholesterol, regardless of body fat (general and abdominal obesity). Regular walking (between 11 km and 16 km per week) during adulthood has been pointed out as effective in the control of different lipid parameters^22^, but previous studies have reported that this protective effect is mainly mediated by modifications in body composition^23^^,^ 24. In our sample, total body and trunk fat were both positively related to CIT (rho=0.36; p-value=0.003 and rho=0.40; p-value=0.002, respectively) and total cholesterol (rho=0.39; p-value=0.003, only trunk fat). Although obesity plays a central role in the development of metabolic and cardiovascular diseases due its pro-inflammatory action^23^^,^ 24, our findings ratify that regular physical activity in daily life (not necessarily physical exercise) can be used as a public health strategy to prevent dyslipidemia in adults^25^, regardless of obesity status. 

Regarding this inflammatory action of obesity on the human body^23^^,^ 24, it has been documented over the last decade that physical activity has significant antioxidant and anti-inflammatory effects^26^. Although less investigated in pediatric populations, this protective effect of exercise can counteract the inflammatory action of the adipose tissue^26 ^in adults, regardless of changes in body composition. Corroborating this idea, a recent study identified that, in obese/overweight adolescents, body fat is related to higher inflammatory status and increased carotid intima-media thickness^13^. At the same time, the same sample of overweight/obese adolescents showed higher cardiorespiratory fitness significantly related to lower inflammation status identified by C-reactive protein and lower carotid intima-media thickness^13^. Therefore, the recent scientific literature has consistently pointed out that, regardless of obesity status, physical activity and cardiorespiratory fitness have a significant role in the control of inflammation and, hence, prevention of atherosclerosis since early age. 

In this sample, early physical activity was consistently related to lower lipid, cardiovascular, and adiposity values. Previous studies have reported that adults engaged in sports activities during childhood and adolescence have a decreased likelihood of reporting dyslipidemia, type 2 diabetes mellitus, and arterial hypertension^15^^,^ 16. There is limited data about the relationship between early physical activity and vessel thickness, but there is a longitudinal relationship between accumulated habitual physical activity throughout life (mainly moderate to high intensity) and lower arterial stiffness among adults^27^. In fact, the impact of current physical activity level on CIT progression is at least in part explained by the reduction in cardiovascular risk factors^14^, and it would be possible to assume that this protective effect is linked to the maintenance of physical activity throughout life^15^^,^ 16 because physical activity is sustained from early age to adulthood^15^. 

In contrast, an unexpected finding caught our attention because, in an additional analysis, the relationship between early physical activity and CIT was simultaneously adjusted by all potential confounders previously used (age, sex, general adiposity, trunk fat) plus current physical activity and, surprisingly, the relationship remained statistically significant (b=-0.17 [95%CI: -0.28; -0.05]; p-value=0.004). This finding seems to show that the relationship between early physical activity and CIT could be supported by other pathways and not just the maintenance from childhood/adolescence to adulthood, as previously hypothesized. 

Given this unexpected finding, a new hypothesis had to be formulated and methodological questions were raised. Regarding the new hypothesis, the possible epigenetic effect of physical activity over risk factors for atherosclerosis (such as blood pressure) was considered. For example, people with polymorphisms related to hypertension development are more exposed to the development of the disease when they have a lower physical activity level, and this pattern has been identified in both childhood and adulthood^28^^,^ 29. From this viewpoint, the new hypothesis raises other relevant questions because it is not clear if this protective effect of physical activity occurs during childhood and remains into adulthood. Therefore, if this independent effect of early physical activity is confirmed, it opens the door to a new line of research, which denotes that the relationship between physical activity and human growth constitutes an area not entirely explored and, hence, not completely understood. 

Limitations should be recognized. The cross-sectional design constitutes the main limitation of the study and, therefore, the development of cohorts analyzing this issue is recommended. Moreover, we suggest that further studies analyzing the maintenance of physical activity should take into account inflammatory biomarkers (TNF-a, interleukin-6, C-reactive protein), other important behavioral risk factors (e.g. smoking, alcohol consumption, diet^30^), and possible polymorphisms. Finally, in this study, current physical activity in the last week was considered as habitual physical activity, but it is not clear how much this specific week represents the pattern of habitual physical activity performed in the last months/years. 

## Conclusions

In summary, it is possible to conclude that early physical activity performed during childhood and adolescence has a significant effect on lipid variables and carotid intima-media thickness in adulthood, regardless of current physical activity. 
